# Precision Pharmacotherapy for Obstructive Sleep Apnea: Matching Targeted Drugs to Patient Endophenotypes

**DOI:** 10.3390/ph19091361

**Published:** 2026-08-28

**Authors:** Jayoung Oh, Ji Ho Choi

**Affiliations:** 1 Department of Otorhinolaryngology–Head and Neck Surgery, Soonchunhyang University Cheonan Hospital, Soonchunhyang University College of Medicine, Cheonan 31151, Republic of Korea; planet90@gmail.com; 2 Department of Otorhinolaryngology–Head and Neck Surgery, Soonchunhyang University Bucheon Hospital, Soonchunhyang University College of Medicine, Bucheon 14584, Republic of Korea

**Keywords:** obstructive sleep apnea, pharmacotherapy, endotype, endophenotype, precision medicine, tirzepatide, AD109, atomoxetine, aroxybutynin, loop gain, arousal threshold, acetazolamide, sultiame, solriamfetol, pitolisant

## Abstract

**Background**: Obstructive sleep apnea (OSA) remains under-treated because continuous positive airway pressure, though efficacious, is frequently limited by poor tolerance and adherence. Long considered a purely anatomical disease refractory to drugs, OSA is now understood as the shared endpoint of four measurable endotypes—a collapsible upper airway, poor pharyngeal dilator-muscle responsiveness, high loop gain, and a low arousal threshold—each a distinct pharmacological target. This review synthesizes the mechanistic rationale and clinical evidence for targeting them. **Methods**: In this narrative review, we searched PubMed/MEDLINE, Embase, and the Cochrane Central Register of Controlled Trials (January 2000 to February 2026) for randomized trials, meta-analyses, and mechanistic endotyping studies, appraising the evidence narratively. **Results**: Tirzepatide became the first drug approved for OSA, acting through weight loss to reduce obesity-related mechanical loading, and the noradrenergic–antimuscarinic combination AD109 met its primary endpoint in a pivotal phase 3 trial by targeting dilator-muscle activity independently of weight. Both, however, used AHI-based endpoints in clinically defined rather than endotype-selected populations; AD109’s placebo-corrected effect was modest (−4.0 events/h), and 21.2% versus 3.1% of participants discontinued for adverse events. They are therefore best described as mechanism-informed rather than endotype-validated. Carbonic anhydrase inhibition with acetazolamide or sultiame and selected sedating agents address ventilatory instability and a low arousal threshold, while solriamfetol and pitolisant relieve residual sleepiness. **Conclusions**: Mechanism-informed pharmacotherapy for OSA is now clinically credible, but scalable bedside endotyping, attention to tolerability, and trait-stratified trials are needed before treatment can be called endotype-guided.

## 1. Introduction

Obstructive sleep apnea (OSA) is characterized by repetitive partial or complete collapse of the pharyngeal airway during sleep, producing intermittent hypoxemia, sympathetic surges, and cortical arousals. Left untreated, it is associated with excessive daytime sleepiness, impaired quality of life, hypertension, metabolic dysregulation, and increased cardiovascular, cerebrovascular, and all-cause mortality risk [1,2,3]. Approximately 936 million adults aged 30–69 years have mild-to-severe OSA worldwide, and 425 million have moderate-to-severe disease, making it one of the most prevalent chronic disorders of adulthood [4].

Continuous positive airway pressure (CPAP) is the recommended first-line therapy for moderate-to-severe disease and reliably abolishes obstructive events during use [5]. Its effectiveness in practice, however, is limited by discomfort, claustrophobia, and the need for nightly application, and a substantial proportion of patients decline treatment at titration or discontinue it within the first years [6,7]. Oral appliances, upper-airway surgery, positional therapy, and hypoglossal-nerve stimulation benefit selected patients but are each constrained by variable efficacy, invasiveness, or cost [1]. As a result, many patients remain inadequately treated, with considerable public-health and economic consequences; the annual cost of undiagnosed OSA in the United States has been estimated at approximately USD 150 billion, compared with an estimated USD 12.4 billion required for diagnosis and treatment [8,9]. These limitations have motivated a longstanding interest in an effective and well-tolerated pharmacological alternative.

For many years, this pharmacological approach yielded limited results. A comprehensive review concluded that the single agents tested—including serotonergic and noradrenergic drugs, cholinergic agents, methylxanthines, and sex hormones—produced only modest and inconsistent effects, and attributed these failures to inadequate animal models, incomplete patient phenotyping, and poorly defined outcome measures [10]. The prevailing view was that OSA is primarily anatomical in origin and therefore largely resistant to drug therapy.

This view has since been revised on two fronts. First, quantitative physiology has established that OSA is not a uniform anatomical disorder but the common endpoint of several measurable pathophysiological traits, or endotypes: a collapsible upper airway, ineffective pharyngeal dilator-muscle responsiveness, high loop gain (unstable ventilatory control), and a low respiratory arousal threshold [11,12]. A clinically relevant abnormality in one or more of the three non-anatomical traits is present in approximately 70% of patients, and because each trait has a distinct neurochemical basis, each represents a potential drug target [11,13]. Second, agents developed on the basis of these mechanisms have succeeded in adequately designed trials. In December 2024, the dual glucose-dependent insulinotropic polypeptide (GIP) and glucagon-like peptide-1 (GLP-1) receptor agonist tirzepatide became the first drug approved for OSA, acting through weight loss to reduce obesity-related mechanical loading of the upper airway [14]. Shortly afterwards, the oral noradrenergic–antimuscarinic combination AD109 (aroxybutynin–atomoxetine), developed to enhance pharyngeal dilator-muscle activity, met its primary endpoint in a large phase 3 trial [15]. Neither program, however, selected or stratified patients by a measured endotype [14,15].

This review is organized around the matching of drugs to endotypes. For each endotype, we consider the mechanistic rationale, the methods used to measure the trait, and the most recent randomized and meta-analytic evidence for the agents that address it, together with wake-promoting therapy for residual sleepiness and the emerging role of combination and precision strategies. Throughout, we emphasize the gap between mechanistic rationale and clinical evidence, as most trait-directed agents have not yet been evaluated in patients selected according to their predominant endotype.

We identified relevant literature by searching PubMed/MEDLINE, Embase, and the Cochrane Central Register of Controlled Trials from January 2000 to February 2026 using terms for obstructive sleep apnea, pharmacotherapy, individual endotypic traits, and specific agents; reference lists and recent reviews were also hand-searched. Priority was given to randomized controlled trials, systematic reviews and meta-analyses, and adult mechanistic endotyping studies, with selective citation of preclinical work when clinically relevant mechanisms were being pursued. As a narrative review, study selection and appraisal were not duplicated against pre-specified criteria, so selection bias cannot be excluded.

## 2. The Endotype Framework of Obstructive Sleep Apnea

**Figure 1:**
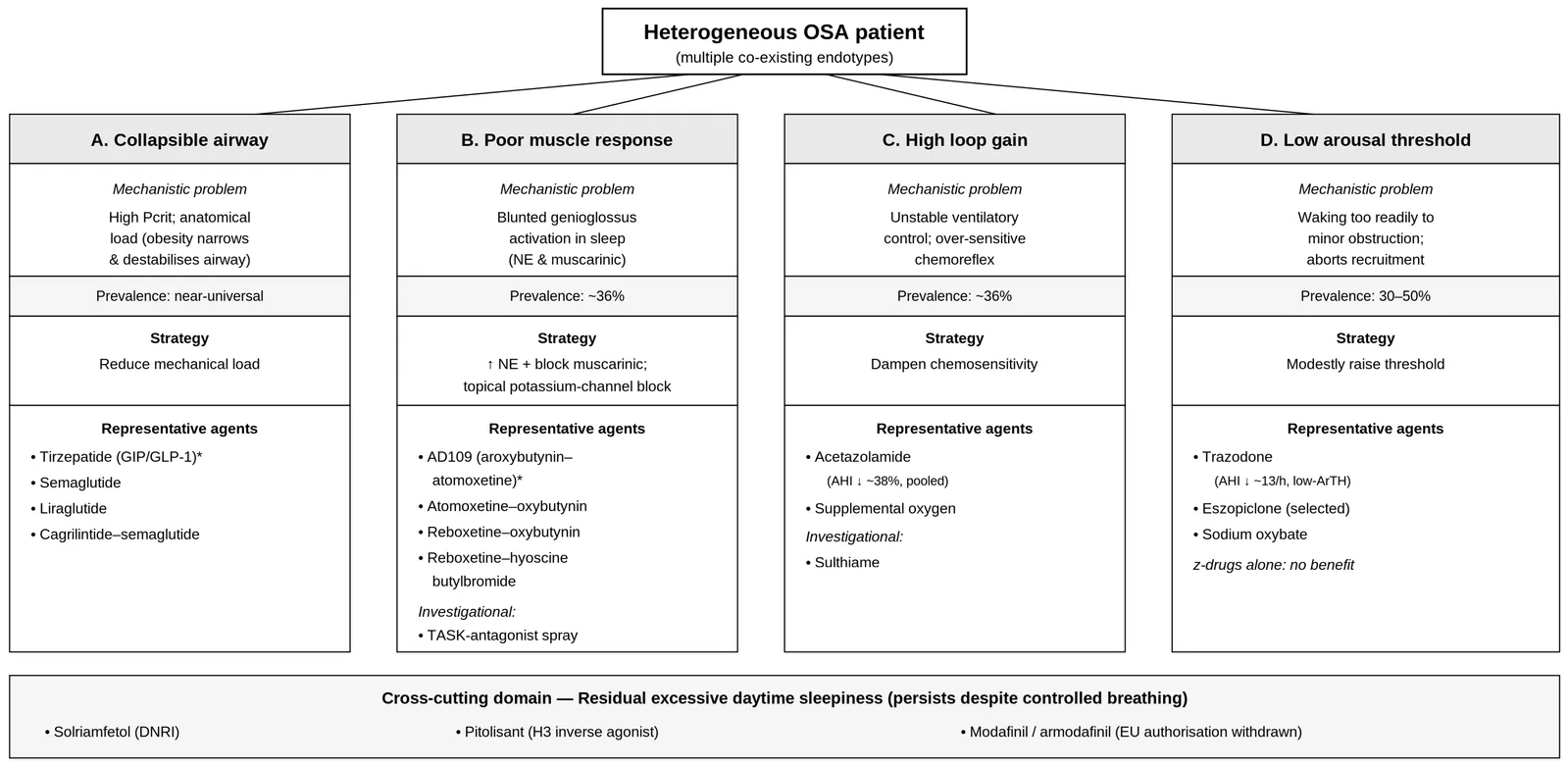
Matching pharmacotherapy to OSA endotypes. The four PALM endotypes and a cross-cutting symptom domain (residual excessive daytime sleepiness), each shown with its mechanistic problem, therapeutic strategy, and representative drug classes. * Agents with regulatory approval (tirzepatide) or a completed pivotal phase 3 trial reported in full (AD109) are marked; neither was evaluated in endotype-selected patients. The figure therefore presents a mechanistic rationale, not a treatment algorithm. *P_crit_, passive critical closing pressure; NE, noradrenaline; ArTH, respiratory arousal threshold; GIP, glucose-dependent insulinotropic polypeptide; GLP-1, glucagon-like peptide-1; DNRI, dopamine–noradrenaline reuptake inhibitor; AHI, apnea–hypopnea index*. ↑, increase; ↓, decrease.

A phenotype classifies patients by observable clinical or physiological features (e.g., sleepiness-predominant, REM-predominant, or positional OSA). An endotype, by contrast, classifies patients by the underlying disease mechanism [16,17]. In an analysis of 75 men and women studied across multiple nights, Eckert et al. quantified four principal traits and formalized the PALM (P_crit_, Arousal threshold, Loop gain, Muscle responsiveness) scale [11]. These traits can now be derived from research polysomnography using controlled CPAP manipulations [12], and increasingly estimated from routine clinical signals [18]. It is important to keep these two tiers of measurement separate. Reference methods quantify each trait directly but require a dedicated research protocol, whereas routine-polysomnography surrogates are indirect proxies that vary widely in how far they have been validated: some have been tested against invasive reference standards, while others remain proposals awaiting formal evaluation. Table 1 sets the two tiers side by side, and Section 10 examines the reliability and limitations of each surrogate in more detail. Figure 1 provides an overview that anchors the remainder of this review, mapping each endotype to its mechanism-matched drug classes.

### 2.1. A Collapsible Upper Airway (Anatomical Compromise)

The tendency of the pharynx to collapse under negative intraluminal pressure is captured by the passive critical closing pressure (P_crit_). A normal airway resists collapse even at sub-atmospheric pressures, whereas an OSA airway may occlude at positive pressures [11]. Impaired anatomy is the single most influential trait and contributes, to varying degrees, in essentially every patient; obesity is a central and modifiable driver because parapharyngeal fat deposition narrows the airway and increases collapsibility [19,20]. This trait is the target of CPAP, oral appliances, surgery and—pharmacologically—of weight-loss therapy.

### 2.2. Poor Upper-Airway Dilator Muscle Responsiveness

During wakefulness, tonic and phasic activity of pharyngeal dilators such as the genioglossus compensates for an anatomically vulnerable airway. In many patients, this reflex responsiveness is blunted during sleep, so the airway is not adequately stiffened as it begins to narrow [11]. Because genioglossus tone is governed largely by noradrenergic excitation (which withdraws at sleep onset) and muscarinic inhibition (prominent in REM sleep), this trait is pharmacologically tractable: augmenting noradrenergic drive while blocking muscarinic inhibition can restore dilator activity [21,22].

### 2.3. High Loop Gain (Unstable Ventilatory Control)

Loop gain is the ratio of a ventilatory response to the disturbance that provoked it. When loop gain is high, small perturbations are over-corrected, and breathing oscillates, driving cyclical airway instability and recurrent events [12]. Lowering chemosensitivity (e.g., with supplemental oxygen) or damping the CO_2_ response (e.g., with carbonic anhydrase inhibition, which produces a mild metabolic acidosis and resets the apneic threshold) can stabilize breathing in these patients [23].

### 2.4. Low Respiratory Arousal Threshold

The respiratory arousal threshold is the level of respiratory drive that triggers cortical arousal. A low threshold—waking too readily to minor airway narrowing—is counter-productive: premature arousal interrupts the accumulation of respiratory stimulus needed to recruit dilator muscles, provokes ventilatory overshoot, and fragments sleep [11]. In appropriately selected patients, agents that modestly raise the threshold can consolidate sleep and reduce event frequency without worsening—and sometimes improving—OSA severity [24,25].

### 2.5. Distribution, Overlap, and Clinical Classification

Most patients have contributions from more than one trait, and a sizeable minority have OSA driven predominantly by non-anatomical mechanisms [11]. Contemporary syntheses estimate that impaired pharyngeal muscle responsiveness and high loop gain each affect roughly a third of patients, and a low arousal threshold affects 30–50% [13,26]. This heterogeneity has two consequences. First, it explains why unselected single-drug trials failed: an agent that corrects one trait appears ineffective when tested in a cohort whose disease is driven mainly by other traits [10,27]. Second, it provides the logic for precision therapy—matching drugs to a patient’s dominant trait, or combining agents to correct several traits at once, and it has become the standard scaffold for cataloging candidate agents by the trait they address [12,28,29].

Two considerations are essential before applying the endotype framework to an individual patient. First, the four traits are interdependent rather than isolated. An intervention aimed at one trait often shifts several others: weight loss reduces P_crit_, but may also lower loop gain and modify the arousal threshold [19,23], whereas noradrenergic–antimuscarinic therapy can improve muscle compensation, collapsibility, loop gain, and the arousal threshold at the same time [30]. Second, the traits differ in how directly they shape the physiological burden of OSA. High loop gain and a low arousal threshold promote ventilatory overshoot, cyclical desaturation, and repeated arousals, thereby contributing to hypoxic burden and sympathetic activation beyond their effect on event counts [31,32]. In practice, a single measured trait rarely explains an individual patient’s OSA, and trait-directed drugs should be expected to produce effects beyond their nominal target. Table 1 and Table 2 summarize the framework: Table 1 by endotype, and Table 2 in the treatment-oriented PALM classification.

The scheme makes the therapeutic logic explicit: patients with a markedly collapsible airway (PALM 1) are best served by anatomical correction or PAP, whereas roughly half of patients with a mildly collapsible or non-collapsible airway (PALM 2b and 3) are those in whom endotype-directed pharmacotherapy is most likely to be of value, alone or as an adjunct [11,27].

## 3. Targeting the Anatomical Endotype: Metabolic Pharmacotherapy

Because obesity increases airway collapsibility, weight reduction directly addresses the dominant anatomical trait. Observational and interventional data show an approximately dose-dependent relationship between weight and OSA severity, with a modest weight change producing a disproportionate change in the apnea–hypopnea index (AHI) [19,20]. Lifestyle intervention alone is frequently insufficient, which historically limited weight loss as an OSA strategy; the advent of highly effective incretin-based anti-obesity drugs has changed this calculus.

### 3.1. Tirzepatide (SURMOUNT-OSA) and the First OSA Drug Approval

Tirzepatide is a once-weekly injectable dual GIP/GLP-1 receptor agonist that suppresses appetite and produces substantial, sustained weight loss. The SURMOUNT-OSA program comprised two 52-week, double-blind, randomized, placebo-controlled phase 3 trials in adults with moderate-to-severe OSA (AHI ≥ 15) and obesity: trial 1 enrolled participants not using PAP and trial 2 those receiving PAP, with participants randomized to the maximum tolerated dose (10 or 15 mg) or placebo [14].

AHI decreased substantially in both trials. In trial 1, it fell by 25.3 events/h with tirzepatide versus 5.3 with placebo; in trial 2, by 29.3 versus 5.5 events/h—treatment differences of −20.0 (95% CI −25.8 to −14.2) and −23.8 events/h, respectively (both *p* < 0.001) [14]. Responder analyses were correspondingly favorable: at week 52, 42.2% of participants in trial 1 and 50.2% in trial 2 met the pre-specified criterion for disease resolution (AHI < 5 events/h, or AHI 5–14 events/h with an Epworth Sleepiness Scale score ≤ 10), compared with 15.9% and 14.3%, respectively, on placebo [14]. Participants lost approximately 18–20% of body weight, and tirzepatide also reduced the sleep apnea-specific hypoxic burden, high-sensitivity C-reactive protein, and systolic blood pressure, and improved sleep-related patient-reported outcomes [14,31]. Adverse events were predominantly gastrointestinal (diarrhea, nausea, vomiting, constipation), generally mild-to-moderate, and most frequent during dose escalation. Based on these data, in December 2024 the U.S. Food and Drug Administration approved tirzepatide as the first pharmacological treatment for moderate-to-severe OSA in adults with obesity, to be used together with a reduced-calorie diet and increased physical activity.

Three qualifications are important when interpreting these findings within the endotype framework. First, the benefit is largely mediated by weight loss and therefore applies most directly to patients with obesity; the trait being modified is fat-related airway loading rather than a neuromuscular or ventilatory deficit [27]. Second, durable benefit is likely to require continued treatment, because weight regain commonly follows discontinuation of anti-obesity pharmacotherapy [19]. Third, SURMOUNT-OSA enrolled participants based on a clinical phenotype—obesity with moderate-to-severe OSA—rather than measured collapsibility or any other endotypic trait. No trait was quantified before or during treatment, and the primary endpoint was the AHI. Moreover, the mechanism is not confined to anatomy, because weight loss can also lower loop gain and modify the arousal threshold [23]. Thus, the trial establishes that a mechanism-informed drug can meaningfully reduce OSA severity, but it does not show that selecting patients by measured endotype improves outcomes [27].

### 3.2. GLP-1 Agonists, Combination Incretins, and Class Effects

Earlier GLP-1 agents provide supporting precedent. In the SCALE Sleep Apnea trial, liraglutide 3.0 mg added to diet and exercise produced a significantly greater reduction in AHI (−12.2 vs. −6.1 events/h; treatment difference −6.1, *p* = 0.015), body weight, and systolic blood pressure than placebo in non-diabetic patients with obesity and moderate-to-severe OSA [33]. Dedicated randomized AHI data for semaglutide remain limited. A systematic review and meta-analysis of GLP-1 receptor agonists in OSA reported significant improvements in AHI, body weight, and systolic blood pressure with a manageable, class-consistent adverse-event profile, though effects on oxygen desaturation index and mean saturation were less consistent [34]. Newer combinations promise still greater weight loss and, potentially, larger anatomical benefit: the amylin-analogue/GLP-1 co-formulation cagrilintide–semaglutide produced additional weight reduction beyond semaglutide alone in phase 3 obesity trials, and its effect on OSA is a logical next question [35]. As a class, incretin-based therapies are likely to become a mainstay for the substantial subgroup of OSA patients in whom obesity is the principal driver, either as monotherapy or in combination with agents addressing coexisting non-anatomical traits [36].

## 4. Targeting Poor Muscle Responsiveness: Noradrenergic–Antimuscarinic Therapy

**Figure 2:**
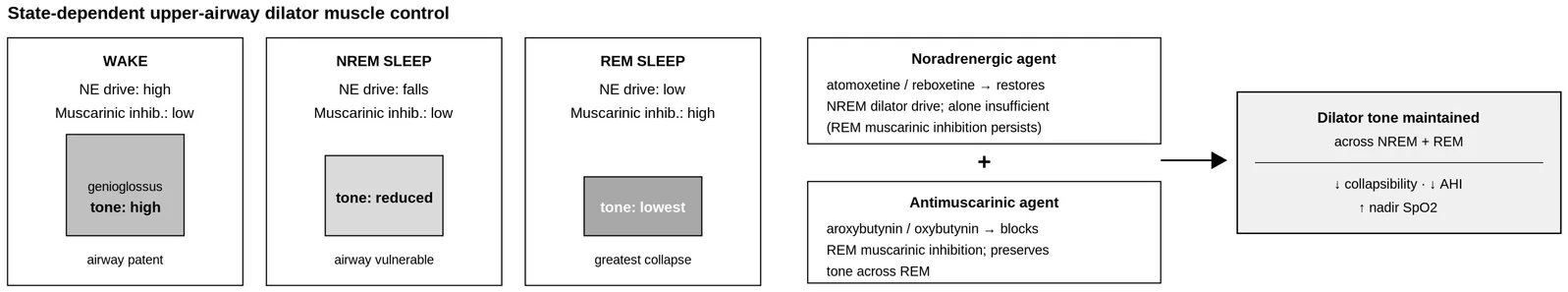
Neurochemical rationale for noradrenergic–antimuscarinic therapy. Noradrenergic (NE) drive falls from wake through NREM sleep, while muscarinic inhibition peaks in REM, producing the greatest loss of genioglossus tone. A noradrenergic agent restores NREM dilator drive but is insufficient alone because REM muscarinic inhibition persists; adding an antimuscarinic preserves tone across REM, reducing collapsibility and OSA severity. *NE, noradrenaline; AHI, apnoea–hypopnoea index; SpO_2_, oxygen saturation.* ↑, increase; ↓, decrease.

Restoring pharyngeal dilator activity during sleep is the most distinctively neuromuscular of the pharmacological strategies, and it applies across weight categories because it corrects a reflex deficit rather than a mechanical load. The physiological logic rests on two opposing neurotransmitter systems (Figure 2). Noradrenaline sustains genioglossus activity during non-REM sleep but withdraws at sleep onset, whereas acetylcholine, acting at muscarinic receptors, drives the profound suppression of genioglossus tone that characterizes REM sleep [21,22,37]. Because dilator tone therefore falls when noradrenergic drive declines and when muscarinic inhibition rises, the rational strategy is to augment noradrenergic transmission while simultaneously blocking muscarinic inhibition. This dual rationale explains why single agents have consistently failed and why fixed noradrenergic–antimuscarinic combinations have succeeded [28,38,39].

### 4.1. Atomoxetine–Oxybutynin: Proof of Concept

The initial proof-of-concept study combined the selective norepinephrine reuptake inhibitor atomoxetine (80 mg) with the antimuscarinic oxybutynin (5 mg), administered once before sleep, in a randomized, placebo-controlled, double-blind crossover trial of 20 patients [38]. A single night of the combination reduced AHI by 63%, from a median of 28.5 to 7.5 events/h, increased nadir oxygen saturation, and approximately tripled genioglossus responsiveness to negative pressure. Neither drug alone reduced AHI, indicating that combined noradrenergic and antimuscarinic action, rather than either mechanism alone, is required to restore upper-airway patency during sleep [38]. This study provided the first demonstration that a rationally selected drug combination can substantially reduce OSA severity.

### 4.2. AD109 (Aroxybutynin–Atomoxetine): Phase 3 Validation

AD109 is a fixed-dose, once-nightly oral combination of aroxybutynin 2.5 mg—the R-enantiomer of oxybutynin, selected for a cleaner anticholinergic profile—and atomoxetine 75 mg [40]. After a positive phase 2b study (MARIPOSA) showing significant AHI improvement over four weeks [41], AD109 advanced to a phase 3 program in adults with mild-to-severe OSA who refused or could not tolerate PAP, comprising the 26-week SynAIRgy and 51-week LunAIRo trials [40]; the completed SynAIRgy trial is the first of these to be reported in full [15].

In SynAIRgy (N = 646 randomized), participants had a median age of 58 years, were 49.3% female, and had a median baseline AHI of 19.6 events/h spanning mild (34.8%), moderate (42.1%), and severe (23.1%) disease—a population reflecting a real-world sleep clinic [15]. The trial met its primary endpoint. In the intention-to-treat analysis, the least-squares mean AHI changed by −3.3 events/h with AD109 and by +0.7 events/h with placebo, giving a placebo-corrected treatment difference at week 26 of −4.0 events/h (95% CI −6.4 to −1.6; *p* = 0.001); expressed as relative change, this corresponds to −44.1% versus −17.6% (*p* < 0.0001) [15]. The absolute effect is thus modest, and for a median baseline AHI of 19.6 events/h it leaves most participants with persistent disease. The on-treatment analysis, restricted to participants still taking the study drug at week 26, yielded larger estimates (−55.6% versus −19.1%; treatment difference −6.5 events/h); because it excludes those who discontinued, it should be read as supportive rather than as the primary result [15].

Oxygenation improved substantially, with significant reductions in the oxygen desaturation index and in hypoxic burden, and 22.3% of on-treatment participants reached disease control (AHI < 5). Benefit was consistent across mild, moderate, and severe OSA and across weight categories, and body-mass index was essentially unchanged, underscoring the weight-independent, neuromuscular mechanism [15]. One methodological limitation was that the patient-reported fatigue endpoint did not reach significance in the intention-to-treat analysis, which broke the hierarchical testing sequence, so downstream secondary endpoints carry only nominal significance [15].

Tolerability is a major qualifier of the SynAIRgy results and should be considered alongside efficacy. Adverse events occurred more often with AD109 than with placebo (70.8% versus 46.7%). More importantly for real-world use, adverse-event-related discontinuation was also substantially higher with AD109 (21.2% versus 3.1%), an approximately sevenfold difference that means about one in five treated participants stopped therapy [15]. The main events—dry mouth, insomnia, nausea, and urinary hesitation—were mechanistically predictable, mostly mild, and tended to occur early after treatment initiation; no treatment-related serious adverse events or deaths were reported [15]. Thus, AD109 is best characterized as a mechanistically effective but only moderately tolerated regimen, and its discontinuation rate is central to estimating the benefit likely to be achieved in routine practice. The two findings should be read together rather than separately: a placebo-corrected reduction of 4.0 events/h delivered to the four in five patients who continue treatment is a different proposition from the same reduction delivered to all who start it, and the trial cannot indicate whether those who discontinued would have been among the responders. In practice this argues for early and explicit review of tolerability—within the first few weeks, when the characteristic antimuscarinic and noradrenergic effects emerge—together with objective confirmation of response before treatment is continued indefinitely, and for counseling patients in advance that roughly one in five will stop for this reason. Section 9 sets out the corresponding cautions and monitoring. As the larger of two registrational trials, SynAIRgy provides the first full peer-reviewed phase 3 evidence that a bedtime oral pill can reduce airway obstruction by targeting a neuromuscular contributor to OSA; the 51-week LunAIRo trial has been described in its design and baseline report [40] and, once reported in full, will define the durability of this effect over twelve months.

Two limitations are central when interpreting SynAIRgy through an endotype framework. First, participants were enrolled based on AHI-defined OSA and PAP intolerance, not on measured pharyngeal dilator-muscle responsiveness. The trial therefore tested a mechanistically rational drug in a broad clinical population rather than validating a strategy in which treatment is assigned according to a documented neuromuscular endotype. Second, endotypic traits were not quantified at baseline or re-assessed during therapy. As a result, the degree to which the observed AHI reduction was mediated by improved dilator-muscle responsiveness cannot be established from SynAIRgy itself and must be inferred from earlier physiological studies [30,38].

### 4.3. Reboxetine-Based and Other Combinations

The success of atomoxetine-based therapy has stimulated exploration of related pairings, summarized with the pivotal trials in Table 3. Reboxetine combined with oxybutynin reduced OSA severity and improved daytime vigilance in a crossover trial [42], and reboxetine combined with the antimuscarinic hyoscine butylbromide likewise lowered AHI and raised nadir oxygen saturation [43]. Atomoxetine has also been paired with fesoterodine in patients with milder collapsibility [44], and comparative work shows that antimuscarinic selectivity matters: non-selective agents (oxybutynin, aroxybutynin) generally outperform more selective compounds such as solifenacin, whereas low-central-penetration agents such as hyoscine butylbromide may retain efficacy with fewer central effects [45]. The single-agent precursor desipramine reduced collapsibility and OSA severity only in patients with minimal muscle compensation, reinforcing that noradrenergic monotherapy is insufficient for most patients [46]. A conceptually important variation replaces the antimuscarinic with a sleep-protecting agent—for example, atomoxetine plus the sedating antidepressant trazodone, or the addition of low-dose zolpidem to atomoxetine–oxybutynin—to counter the tendency of a purely noradrenergic–antimuscarinic combination to lower the arousal threshold [47,48].

Pooled analyses indicate that the benefit reflects the drug combination’s mechanism rather than a property of any single agent. A 2022 meta-analysis found that noradrenergic–antimuscarinic therapy reduced AHI and improved oxygenation across a range of drug pairings [49]. A subsequent meta-analysis of eight crossover RCTs (n = 177) reported a pooled AHI reduction of 9.0 events/h and a 5.6-percentage-point improvement in the lowest oxygen saturation, and identified sex as the only significant effect modifier, with a higher proportion of male participants associated with greater AHI reduction, possibly because endogenous progesterone enhances genioglossus tone in women [50]. The most comprehensive analysis to date, comprising thirteen RCTs (n = 345), confirmed significant reductions in AHI by both 3% and 4% desaturation criteria (mean differences −6.30 and −6.50 events/h) and showed that the combined regimen improves nearly every measurable endotypic trait, including loop gain, pharyngeal muscle compensation and recruitment, collapsibility, and the arousal threshold [30]. This trait-level evidence that a single strategy modifies several endotypes in the intended direction provides mechanistic support for the endotype framework. One safety observation from the same analysis is relevant: the regimen increased resting heart rate by approximately 7 beats per minute, attributable to the noradrenergic component, without a significant change in blood pressure, so long-term cardiovascular safety in older patients requires further study [30].

### 4.4. From Neurochemistry to Druggable Targets: The Hypoglossal Motor Nucleus

The clinical success of noradrenergic–antimuscarinic therapy rests on a specific neuroanatomical substrate: the hypoglossal motor nucleus (HMN) and its premotor circuitry, which govern the genioglossus and other upper-airway dilators. Genome-wide profiling of the HMN has identified a set of enriched, druggable modulators of motoneuron activity, among them the α1a-adrenergic receptor and the motoneuron-selective potassium channels Kir2.4 and TASK [51,52], and chemogenetic studies have shown that selectively activating hypoglossal motoneurons sustains tongue-muscle activity across sleep and enlarges the upper airway without disturbing sleep–wake state [53]. The same channel family has proved tractable at a different anatomical site: a TASK-channel antagonist nasal spray (BAY2586116), which acts locally on upper-airway mechanoreceptors rather than centrally on motoneurons, increased pharyngeal dilator muscle activity, reduced collapsibility, and lowered OSA severity in participants who showed a physiological reduction in P_crit_, with minimal systemic exposure [54]. Topical potassium-channel blockade therefore offers a side-effect-sparing route to the muscle-responsiveness endotype, achieving a reduction in collapsibility by restoring dilator activity rather than by altering anatomy [52].

## 5. Targeting High Loop Gain: Ventilatory Control Stabilizers

### 5.1. Acetazolamide

Acetazolamide is a carbonic anhydrase inhibitor that induces a mild metabolic acidosis, increasing resting ventilation and resetting the CO_2_ (apneic) threshold, thereby reducing loop gain and stabilizing breathing [23]. A comprehensive systematic review and meta-analysis found that acetazolamide reduced AHI by approximately 38% (about −13.8 events/h), with a greater effect at higher doses and improvement in nadir oxygen saturation [55]. Mechanistic meta-analysis confirms that the drug lowers loop gain and that the reduction in loop gain correlates with the reduction in AHI [23,56]. Acetazolamide is particularly attractive for patients whose OSA is driven by ventilatory instability and for periodic breathing at altitude, though its tolerability is dose-limited: paresthesia, altered taste, metabolic acidosis, hypokalemia, and an increased risk of nephrolithiasis can occur, and these are considered further in Section 9. Its principal limitation is shared with all single agents: efficacy depends on selecting patients in whom high loop gain is a dominant trait [10].

### 5.2. Sultiame (The FLOW Study)

Sultiame (sulthiame) is a sulfonamide carbonic anhydrase inhibitor long used for childhood focal epilepsy and now repurposed for OSA. Early randomized data established acceptable safety and tolerability while suggesting efficacy in sleep apnea [57]. Mechanistically, sultiame resembles acetazolamide by inducing mild metabolic acidosis and stabilizing ventilatory control, but it appears to have broader endotypic effects. Dedicated physiological work showed reductions in loop gain and collapsibility together with an increase in the arousal threshold, suggesting actions beyond a purely chemoreceptor-directed mechanism [58].

The phase 2 FLOW study is the largest pharmacological trial reported in OSA to date. In this 15-week, multicenter, randomized, placebo-controlled dose-finding trial, 298 adults with untreated moderate-to-severe OSA were assigned to placebo or once-nightly sultiame 100, 200, or 300 mg across 28 European sites [59]. At week 15, placebo-subtracted relative reductions in AHI_3a_ were −16.4%, −30.2%, and −34.6% for the 100, 200, and 300 mg doses, respectively, with parallel improvements in nocturnal oxygenation, sleep quality, and daytime sleepiness [59]. Adverse events increased dose-dependently, mainly because of paresthesia, and only 240 of 298 participants completed treatment; the investigators therefore judged 200 mg to offer the most favorable efficacy–tolerability balance [59].

Sultiame and acetazolamide share carbonic anhydrase inhibition, loop-gain reduction, and dose-related paresthesia or acid–base disturbance, but they should not be treated as interchangeable. Acetazolamide has a larger pooled reported AHI effect, approximately −38%, but this estimate comes from small, short, heterogeneous studies and the drug has no OSA registration pathway [55]. Sultiame has a smaller but prospectively measured effect from an adequately powered dose-finding trial, possible additional actions on upper-airway muscle function, and an active development program. Sultiame nevertheless remains a phase 2 agent: follow-up is limited to 15 weeks, no direct comparison with acetazolamide has been performed, and—critically—it has not been tested in patients selected for high loop gain [27,59].

### 5.3. Supplemental Oxygen and Oxygen–Hypnotic Combinations

Supplemental oxygen is not a pharmacological agent in the strict sense, and it therefore falls outside the scope of a review of pharmacotherapy. We retain a short account of it here for two reasons: it is the most direct available means of lowering loop gain, and it is the comparator against which chemoreceptor-directed drugs are judged. Supplemental oxygen lowers loop gain by reducing peripheral chemosensitivity to CO_2_, damping ventilatory overshoot. As monotherapy, its effect on obstructive events is modest and inconsistent, and it can lengthen apneas by blunting the hypoxic drive to arouse [10]. However, because oxygen and sedative–hypnotics act on complementary traits—oxygen on loop gain and a hypnotic on the arousal threshold—their combination is mechanistically appealing, and early physiological work suggests that rationally combining two trait-directed interventions can benefit patients who respond poorly to either alone [23].

## 6. Targeting the Low Arousal Threshold: Sedative–Hypnotics

As previously described, modestly raising an inappropriately low arousal threshold can improve OSA rather than worsen it [24,25]. The key requirement is to consolidate sleep without depressing dilator tone or ventilatory drive to a degree that worsens obstruction, so agent selection and patient selection are both critical.

Trazodone, a serotonin antagonist and reuptake inhibitor, is the best-characterized agent: it raised the respiratory arousal threshold in patients with a low threshold without abolishing dilator responsiveness, and reduced AHI in subsequent work [25,60]. Eszopiclone increased the arousal threshold and lowered AHI specifically in the low-arousal-threshold subgroup [24]. By contrast, a systematic review and meta-analysis of hypnotics found that zolpidem, zopiclone, and eszopiclone did not consistently reduce AHI, and cautioned against hypnotic monotherapy except in carefully selected patients [61]. A broader meta-analysis of OSA pharmacotherapy that pooled 29 studies across 11 drugs reinforced this pattern: trazodone produced the largest AHI reduction among arousal-directed agents (mean difference approximately −12.8 events/h), with significant but smaller effects for donepezil (acting on loop gain) and sodium oxybate, whereas benzodiazepines and z-drugs showed no consistent benefit [62]. Recent combinations—viloxazine with trazodone, and atomoxetine with trazodone—extend the design principle of pairing a dilator-muscle activator with a sleep-protecting agent [47,63,64]. Table 4 summarizes these agents. Safety deserves particular emphasis for this class, because the patients in whom these drugs would be used overlap substantially with those most vulnerable to their harms; the specific risks and cautions are set out in Section 9.

## 7. Adjunctive Pharmacotherapy for Residual Excessive Daytime Sleepiness

The endotype-directed strategies above aim to reduce obstructive events. However, effective control of breathing does not always abolish the daytime consequences: a clinically important minority of patients remain sleepy despite adequate, well-adherent therapy, once poor adherence and other sleep disorders are excluded [65]. For these patients, wake-promoting pharmacotherapy is a complementary, symptom-directed axis that is likely to remain relevant even as endotype-targeted drugs reduce the underlying event burden.

Historically, residual excessive daytime sleepiness (EDS) was treated with sympathomimetic psychostimulants and, later, with modafinil and armodafinil, which improve wakefulness in CPAP-treated patients without reducing apneas; the European marketing authorization of modafinil for OSA was subsequently withdrawn on benefit–risk grounds, and abuse potential remains a consideration [9,65]. Two mechanistically novel agents have since expanded the options. Solriamfetol, a selective dopamine–norepinephrine reuptake inhibitor that lacks the monoamine-releasing effect of amphetamines, produced dose-dependent improvements in objective wakefulness and subjective sleepiness in the phase 3 TONES 3 trial and is approved by both the FDA and the EMA [66]. Pitolisant, a histamine H_3_-receptor inverse agonist, reduced residual EDS in CPAP-adherent and CPAP-refusing patients in the HAROSA program, with a favorable safety profile and no appreciable abuse liability [67,68]. Both agents explicitly do not treat the airway obstruction and are not substitutes for primary therapy. Indirect comparisons suggest broadly similar symptomatic benefit, allowing selection to be guided by tolerability, comorbidity, and abuse-liability considerations [69]. Table 5 summarizes these wake-promoting agents alongside the breathing-directed classes.

## 8. From Single Agents to Combination and Precision Therapy

**Figure 3:**
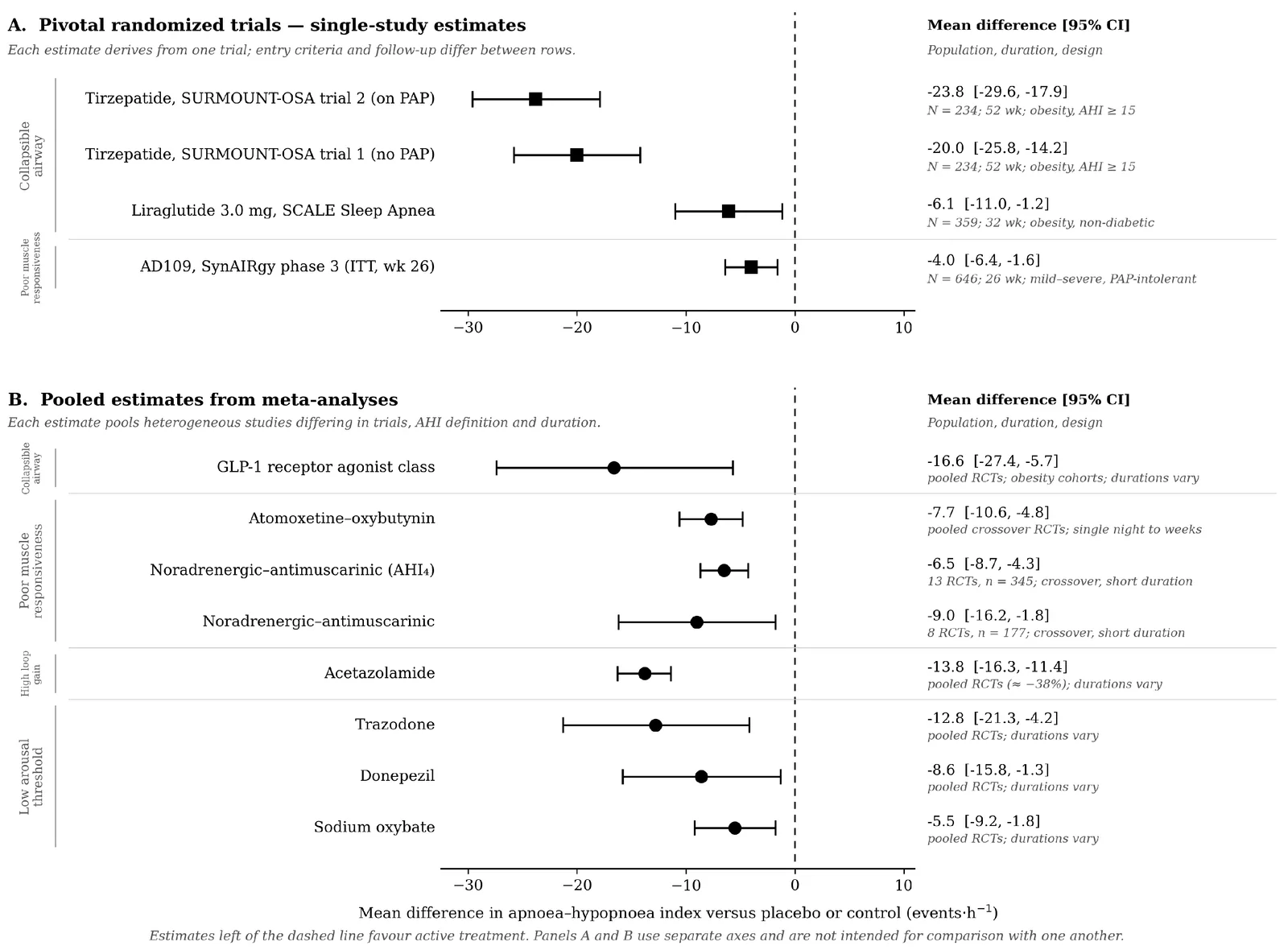
AHI reduction reported for endotype-targeted drug classes, grouped by evidence type: (**A**) pivotal randomized trials and (**B**) pooled meta-analytic estimates. Rows are organized by the intended endotype, with population, duration, and study design shown for context. Square markers indicate single-trial estimates, circles indicate pooled estimates, and horizontal bars show 95% confidence intervals. Because studies differed in population, AHI definition, regimen, and duration, the figure should be read as a summary of reported effect ranges, not as a ranking of drug classes; formal comparison would require a network meta-analysis. *AHI, apnea–hypopnea index; PAP, positive airway pressure; ITT, intention-to-treat.*

The benefit of any single agent depends on whether the treated patient’s disease is driven by the trait that the drug addresses [10,27,28]. The recent successes fit this pattern: tirzepatide addressed a near-universal contributor (obesity), and AD109 acted on a mechanism likely to be relevant across a broad clinical population (dilator-muscle activity) [14,15]. Figure 3 summarizes the AHI reductions reported for each endotype-targeted class, with pivotal single-trial and pooled meta-analytic estimates shown in separate panels. The largest effects are those of the weight-directed agents, which were tested in populations enrolled on the basis of obesity, whereas the remaining estimates derive largely from trials that did not select patients by endotype. This pattern also implies that the greatest gains will come from two complementary directions: matching a drug to a patient’s dominant endotype, and combining agents to correct several traits at once. Combination approaches are already emerging, as with the oxygen–eszopiclone and atomoxetine–oxybutynin–zolpidem regimens noted above [23,48]. Looking further ahead, patients with obesity-driven OSA and a coexisting neuromuscular deficit might benefit from an incretin agent combined with a neuromuscular modulator—a hypothesis that follows directly from the endotype framework and merits formal testing [28,36].

In practice, OSA drugs may be most useful as add-ons to established anatomical therapies rather than as stand-alone replacements. This is the PALM 2b scenario: a narrow but not severely collapsible airway is partially corrected by PAP, an oral appliance, or surgery, but residual disease persists because a non-anatomical trait remains untreated [11]. Three examples illustrate this strategy. First, hypnotics may improve early PAP acclimatization when a low arousal threshold contributes to intolerance; in a systematic review and meta-analysis, non-benzodiazepine sedative–hypnotics—especially eszopiclone—improved CPAP adherence, whereas evidence for zolpidem and zaleplon was insufficient [70]. Second, combining a mandibular advancement device with supplemental oxygen addresses both collapsibility and ventilatory instability, and a randomized trial showed better outcomes than either intervention alone [71]. Third, add-on pharmacotherapy may also help after upper-airway surgery, where residual OSA can reflect persistent non-anatomical traits; this rationale underpinned a double-blind randomized trial of acetazolamide after barbed reposition pharyngoplasty [72]. The adjunctive approach also has a practical dosing advantage: because the drug targets residual physiology rather than the entire disorder, lower doses may be adequate—an important consideration given tolerability limits with both incretin and noradrenergic–antimuscarinic therapy. Trait-stratified trials of drug–device and drug–surgery combinations are therefore among the most feasible next steps for precision OSA pharmacotherapy.

## 9. Safety, Tolerability, and Contraindications

Because these agents would be taken nightly or weekly for years by people who are frequently middle-aged, overweight, and comorbid, tolerability and safety are as decisive as efficacy, and several class-specific concerns deserve explicit statement. Sedative–hypnotics increase the risk of falls and fractures in older adults, may produce next-day sedation with impaired driving performance, and can worsen obstruction where respiratory drive or dilator tone is suppressed; they should be avoided in patients with an already high arousal threshold, significant hypercapnia, or concomitant opioid or alcohol use [24,61]. Incretin therapies cause gastrointestinal intolerance during dose escalation and produce loss of lean as well as fat mass, raising the question of sarcopenia in older patients, and they are contraindicated in medullary thyroid carcinoma, multiple endocrine neoplasia type 2, and prior pancreatitis, with weight regain expected after discontinuation [14,19]. Carbonic anhydrase inhibitors cause paresthesia, metabolic acidosis, hypokalemia, and an increased risk of nephrolithiasis, and should be avoided in significant renal or hepatic impairment and in sulfonamide hypersensitivity [55,59]. Noradrenergic–antimuscarinic combinations raise resting heart rate by approximately 7 beats per minute and cause dry mouth and urinary hesitation, so caution is warranted in cardiovascular disease, narrow-angle glaucoma, and bladder outlet obstruction, and roughly one in five participants discontinued AD109 for this reason in SynAIRgy [15,30]. Wake-promoting agents raise blood pressure and heart rate (solriamfetol) or prolong the QT interval (pitolisant) [66,67]. Table 6 summarizes these considerations.

Because these are chronic therapies, the most important safety questions concern cumulative cardiovascular risk, and the evidence base is at present much shorter than the intended duration of treatment. For noradrenergic–antimuscarinic therapy the longest completed trial is 26 weeks, and the 51-week LunAIRo trial, once reported in full, will provide the first year-long data on heart rate and blood pressure during continuous use [15,40]; given the observed rise in resting heart rate of approximately 7 beats per minute, ambulatory blood-pressure and heart-rate monitoring, together with systematic capture of arrhythmia and major adverse cardiovascular events, would be a reasonable minimum for future trials and post-marketing surveillance, particularly in older patients and in those with established cardiovascular disease. The position for incretins is more favorable, since cardiovascular outcome trials in overweight and obese populations without diabetes have already shown a reduction in major adverse cardiovascular events with semaglutide [73]; what remains unresolved for OSA specifically is whether the loss of lean mass matters over years, and what happens to both weight and apnea severity after treatment stops. For carbonic anhydrase inhibitors, no long-term OSA safety data exist beyond 15 weeks, so periodic monitoring of electrolytes, bicarbonate, and renal function is prudent if treatment is continued [59]. Linking these agents to existing sleep-apnea and obesity registries, and adding cardiovascular endpoints to endotype-stratified trials, would generate this evidence faster than dedicated safety trials alone.

## 10. Toward Clinical Implementation

Before endotype-guided pharmacotherapy can be implemented clinically, a major measurement gap must be addressed. The reference methods for defining OSA endotypes depend on labor-intensive research physiology, including repeated CPAP dial-down and dial-up maneuvers during attended polysomnography to derive P_crit_, ventilatory response, genioglossus responsiveness, and respiratory arousal threshold [11,12]. These protocols often require a dedicated research night, trained technical support, esophageal or epiglottic pressure catheters or intramuscular electromyography, and off-line signal analysis. Because such procedures are not part of routine sleep-laboratory practice—and are unlikely to become so at scale—the key implementation question is not whether clinically meaningful endotypes exist, but whether they can be estimated accurately enough from the diagnostic polysomnogram already obtained in usual care. Bridging this gap is an active priority, and a 2025 American Thoracic Society research statement set out the agenda for translating endophenotyping to the clinic [18]. Several candidate surrogates can be derived from routine polysomnography, although they differ considerably in how far they have been validated: a high apnea-to-hypopnea ratio and a higher therapeutic CPAP requirement indicate a more collapsible airway; a validated multi-variable score (e.g., AHI < 30 events/h, higher nadir SpO_2_, and a high hypopnea fraction) identifies a low arousal threshold; progressive flow limitation within events has been proposed as an indicator of poor dilator compensation, although it currently depends on research-grade flow-shape analysis rather than standard scoring; and cyclical breathing with prominent central events suggests high loop gain [18,26]. Table 1 contrasts the reference techniques with these routine-PSG surrogates. Their validation status differs substantially. The multi-variable arousal-threshold score is the best characterized: it was derived and validated against esophageal-pressure measurement in physiologically phenotyped cohorts and classifies a low arousal threshold with useful, though imperfect, accuracy, so it is best used to enrich for a trait rather than to confirm it in an individual. The loop-gain signals are recognized qualitatively rather than by a validated threshold; the collapsibility proxies have face validity, but no agreed cut-off; and the flow-limitation marker of dilator compensation has not been prospectively validated against genioglossus electromyography in routine practice [18,26]. A realistic near-term pathway is to use such surrogates to identify candidates most likely to respond to a given trait-directed drug and to confirm response objectively with a follow-up sleep study.

The currently available surrogates are not equally mature, and this unevenness should temper expectations for bedside endotyping. The most clinically usable signals are those for a low arousal threshold and high loop gain: the multi-variable arousal-threshold score has been validated against esophageal pressure, while cyclical crescendo–decrescendo breathing, treatment-emergent central events, and a high AHI in a non-obese patient can be recognized in routine reports [18]. By contrast, collapsibility and pharyngeal dilator-muscle responsiveness remain difficult to infer. Candidate markers of Pcrit, such as the apnea-to-hypopnea ratio and therapeutic CPAP requirement, are confounded by body position, sleep stage, arousal, and night-to-night variability. Muscle responsiveness is even less clinically accessible: flow-shape analysis is promising, but it remains research software rather than a standard scored PSG variable [18]. In routine practice, therefore, clinicians can reasonably suspect a low arousal threshold or ventilatory instability from a standard study, but they cannot yet reliably identify the patient whose OSA is driven primarily by impaired muscle compensation—the group in whom noradrenergic–antimuscarinic therapy should, in principle, work best. For a clinician reading a standard report today, the most practical starting point is therefore a small set of variables that every laboratory already scores: the AHI itself, the hypopnea-to-apnea balance, the nadir and pattern of oxygen desaturation, and the distribution of events by sleep stage and body position. A patient with an AHI below 30 events/h, a high hypopnea fraction, and a relatively preserved nadir SpO2 is likely to have a low arousal threshold; one with cyclical breathing, treatment-emergent central events, or a high AHI despite a normal body-mass index is more likely to have high loop gain; and marked supine or REM predominance points to an anatomical contribution. These are enrichment signals, not diagnostic tests, and they should be used to weight a treatment choice rather than to assign a patient to an endotype. Until more robust tools are available, the practical model is an explicit therapeutic trial: select patients using the best available surrogate, initiate treatment, and verify response objectively rather than assume it. Given substantial night-to-night variability in AHI, repeated home sleep testing before and after drug initiation is likely to become the pragmatic standard for judging response, and scalable diagnostic tools remain the rate-limiting step for routine endotype-guided pharmacotherapy [18].

On this basis, a provisional sequence can be proposed, offered as a framework for discussion rather than a validated algorithm. (i) Confirm that the diagnosis and any anatomical therapy have been optimized, since pharmacotherapy is at present an adjunct rather than a replacement. (ii) Review the routine study for the enrichment signals above and identify the trait most likely to be dominant. (iii) Where obesity is present, address it first, since weight loss modifies several traits at once and has the largest reported effect. (iv) Otherwise, match the drug class to the suspected trait—a noradrenergic–antimuscarinic combination where dilator-muscle compensation appears to fail, a carbonic anhydrase inhibitor where breathing is unstable, a sedating agent where the arousal threshold appears low—while checking the cautions in Table 6. (v) Re-measure with a repeat sleep study after an adequate trial, and stop the drug if the response is absent. (vi) Consider a rational combination only when a single agent has produced a partial response. Each step rests on inference from surrogates rather than on measured traits, and the sequence should be tested prospectively before it is recommended for practice.

Several other issues will shape adoption. Regulatory endpoints have historically centered on AHI, yet AHI correlates only moderately with the cardiovascular and neurocognitive sequelae that matter most; measures such as hypoxic burden—which predicts cardiovascular mortality—together with patient-reported outcomes should be incorporated into future trials [31,32]. Long-term safety and durability data remain limited for the newest agents, and adherence to a nightly or weekly pharmacotherapy—while likely better than to PAP—needs real-world confirmation. Finally, cost, insurance coverage, and equitable access, particularly for high-cost incretin therapies, will strongly influence who actually benefits, a consideration that must be weighed against the large economic burden of untreated disease [8,9].

## 11. Discussion: Current Evidence and Limitations

The advances summarized here are substantive, but they should be interpreted alongside a critical-appraisal literature that qualifies current expectations. A 2025 critical review of randomized placebo-controlled trials found that most endotype-targeted agents have not been evaluated in endotype-selected samples; that in several trials the reduction in AHI did not correlate with a change in the physiological trait the drug was intended to modify; and that weight-loss therapy, principally tirzepatide, remains the only pharmacological strategy with sufficient evidence for routine clinical use, with other agents showing modest and inconsistent effects and, in some cases, adverse effects that oppose the therapeutic goal [27,74]. These observations do not invalidate the endotype paradigm; rather, they define its unfinished agenda. The positive AD109 phase 3 trial provides some of the strongest evidence that a rationally targeted, weight-independent mechanism can produce a statistically robust—if absolutely modest—reduction in OSA severity [15], and the trait-level meta-analysis showing that noradrenergic–antimuscarinic therapy improves several endotypes concurrently supports the underlying physiology [30].

The distinction between mechanism-informed and endotype-guided treatment is central to interpreting the current field. SURMOUNT-OSA and SynAIRgy were landmark studies because both showed that drugs selected on mechanistic grounds can reduce AHI in broad OSA populations [14,15]. However, neither trial measured the endotype that the intervention was presumed to modify. Participants were selected by AHI and clinical characteristics; AHI served as the primary endpoint, and no physiological trait was quantified at baseline or re-measured during treatment. These trials therefore support mechanism-informed pharmacotherapy, but they do not yet establish the stronger claim required for endotype-guided care: that measuring a trait and assigning therapy accordingly improves outcomes compared with treating without such measurement. To date, genuinely trait-selected evidence remains limited to small, short physiological studies, including eszopiclone in patients with a low arousal threshold and a TASK-channel antagonist spray in physiological responders [24,54]. Thus, the evidence base is best described as phase 3-supported mechanism-informed pharmacotherapy, whereas true endotype-guided pharmacotherapy remains supported mainly by mechanistic physiology rather than by prospective outcome trials.

A further lesson concerns evidence quality. Much of the endotype-targeted literature derives from small, single-night or few-week crossover studies; only tirzepatide (52 weeks) and AD109 (26 weeks, with a 51-week trial pending full publication) rest on long-duration, adequately powered trials [14,15,27]. As the field matures, the priority is to move from physiological proof-of-concept to pragmatic, endpoint-diverse, endotype-stratified trials that report cardiovascular and patient-centered outcomes alongside AHI.

## 12. Conclusions

OSA pharmacotherapy has changed markedly in a short time. A field long defined by unselected trials of single agents now has an approved drug and a second compound with positive phase 3 results, both developed from an explicit mechanistic rationale [14,15]. The endotype framework explains both halves of that history—why the earlier agents failed, and why these two succeeded—but it has not yet changed how patients are chosen: prescribing remains mechanism-informed rather than guided by a measured endotype. Both agents also carry tolerability costs that will shape how widely they are used. Further progress in endotype-guided, combination, and individualized pharmacotherapy will depend on several developments: (i) validated, scalable tools to determine endotypes from routine clinical data; (ii) prospective, endotype-stratified trials of rational combinations, such as an incretin with a neuromuscular modulator or a muscle activator with a loop-gain stabilizer; (iii) endpoints beyond AHI, including hypoxic burden, blood pressure, cardiovascular events, and quality of life; (iv) long-term durability and safety data, including the cardiovascular safety of chronic noradrenergic therapy and weight regain after incretin discontinuation; (v) strategies to contain cost and improve access, particularly for incretin therapies; and (vi) continued development of novel targets such as the HMN potassium channels and orexinergic modulators [18,52,54,75]. OSA is a heterogeneous disorder, and effective pharmacotherapy will require matching the mechanism of action to the individual patient.

## Figures and Tables

**Table 1:** OSA endotypes: reference measurement methods, surrogate markers obtainable from routine clinical polysomnography, and representative pharmacological agents.

Endotype (Approx. Prevalence)	Reference Measurement (Research)	Surrogate from Routine Clinical PSG	Representative Agents (Evidence Status)
A. Collapsible upper airway (near-universal)	Passive P_crit_ from CPAP dial-down; ventilation at eupnoeic pressure	High apnea-to-hypopnea ratio; high therapeutic CPAP requirement; low nadir SpO_2_; supine predominance	Tirzepatide; semaglutide; liraglutide; cagrilintide–semaglutide (phase 3 positive; tirzepatide FDA-approved, obesity phenotype not endotype-selected)
B. Poor muscle responsiveness (~36%)	Genioglossus EMG response to negative pressure or to CPAP dial-down	Progressive flow limitation within events; failure of events to self-terminate; limited compensation on flow-shape analysis (research software)	AD109 (aroxybutynin–atomoxetine); atomoxetine–oxybutynin; reboxetine combinations; TASK-channel antagonist spray (phase 3 positive but not endotype-selected; spray tested in physiological responders)
C. High loop gain (~36%)	Steady-state or CPAP dial-up ventilatory response; model-derived loop gain	Cyclical crescendo–decrescendo breathing; coexisting central or mixed events; treatment-emergent central apnea; high AHI with low BMI	Acetazolamide; sultiame (investigational); supplemental oxygen (not a drug) (phase 2 dose-finding for sultiame; small RCTs and meta-analysis for acetazolamide; no endotype-selected trial)
D. Low arousal threshold (30–50%)	Negative esophageal or epiglottic pressure at the arousal breath	Validated multi-variable score (AHI < 30 events/h, higher nadir SpO_2_, high hypopnea fraction); short arousal-to-event latency	Trazodone; eszopiclone; sodium oxybate (small RCTs; eszopiclone is the only agent tested prospectively in a trait-selected subgroup)

Prevalence estimates are approximate and derived from cited physiological cohorts and syntheses. Surrogate markers are indicative only and have been validated to differing degrees; those for collapsibility and for dilator-muscle responsiveness are the least well established (see Section 10). *P_crit_, passive critical closing pressure; PSG, polysomnography; CPAP, continuous positive airway pressure; EMG, electromyography; AHI, apnea–hypopnea index; RCT, randomized controlled trial.*

**Table 2:** PALM classification of OSA patients by airway collapsibility, with the observed distribution and implied treatment strategy.

PALM Group	Patients	P_crit_/Defining Feature	Implied Treatment Strategy
PALM 1	~23%	P_crit_ ≥ +2 cmH_2_O; highly collapsible airway	Aggressive anatomical correction or PAP; pharmacotherapy alone unlikely to suffice
PALM 2a	~21%	P_crit_ −2 to +2 cmH_2_O; narrow airway, no major non-anatomical trait	Anatomical correction (PAP, oral appliance, surgery, weight loss)
PALM 2b	~27%	P_crit_ −2 to +2 cmH_2_O; coexisting non-anatomical trait(s)	Anatomical correction plus endotype-directed pharmacotherapy
PALM 3	~19%	P_crit_ < −2 cmH_2_O; airway not easily collapsible	Endotype-directed pharmacotherapy (muscle, loop gain, or arousal threshold)

Prevalence estimates are based on the physiological cohort reported by Eckert et al. [11]. Non-anatomical traits comprise low arousal threshold, high loop gain, and poor muscle responsiveness. *PAP, positive airway pressure; P_crit_, passive critical closing pressure.*

**Table 3:** Representative randomized trials of noradrenergic–antimuscarinic and related upper-airway neuromodulatory therapy.

Regimen (Dose)	Design/N	Duration	Key AHI Effect	Reference
Atomoxetine 80 mg + oxybutynin 5 mg	Crossover; N = 20	1 night	28.5 → 7.5 events/h (−63%); neither drug alone effective	[38]
Aroxybutynin 2.5 mg + atomoxetine 75 mg (AD109)	RCT; N = 646	26 wk	−44.1% vs. −17.6% placebo (ITT); disease control 22.3%	[15]
Aroxybutynin 2.5 mg + atomoxetine 75 mg (AD109)	RCT; N = 211	4 wk	AHI_4_ −47.1% (2.5/75 mg dose) vs. placebo	[41]
Reboxetine 4 mg + oxybutynin 5 mg	Crossover; N = 16	1 wk	Reduced AHI and improved oxygenation vs. placebo	[42]
Reboxetine 4 mg + hyoscine butylbromide 20 mg	Crossover; N = 12	1 night	Reduced AHI; ↑ nadir SpO_2_; ↑ genioglossus tone	[43]
Atomoxetine 80 mg + fesoterodine 4 mg	Crossover; N = 12	1 night	Reduced apnea index; milder-collapsibility responders	[44]
Desipramine 200 mg	Crossover; N = 14	1 night	Improved collapsibility; benefit in minimal-compensation subgroup	[46]

Values are as reported in the cited primary studies; AHI definitions and durations differ. *AHI, apnea–hypopnea index; ITT, intention-to-treat; RCT, randomized controlled trial. ↑, increase.*

**Table 4:** Loop-gain and arousal-threshold agents: mechanism, representative effect, and the endotype for which each is best suited.

Agent (Dose)	Class/Mechanism	Representative Effect	Best-Suited Endotype	Reference
Acetazolamide (250–1000 mg)	CA inhibitor; ↓ loop gain	AHI −38% (pooled); ↓ loop gain ~41%	High loop gain	[23,55]
Supplemental O_2_	↓ peripheral chemosensitivity	Modest/inconsistent AHI effect; better combined	High loop gain	[10,23]
Trazodone (100 mg)	SARI; ↑ ArTH	AHI ~−12.8 events/h (pooled)	Low arousal threshold	[25,62]
Eszopiclone (3 mg)	Non-benzodiazepine; ↑ ArTH	↑ ArTH and ↓ AHI in low-ArTH subgroup	Low arousal threshold	[24]
Sodium oxybate	GABA-B agonist	AHI ~−5.5 events/h (pooled)	Low arousal threshold	[62]
Zolpidem/zopiclone	Non-benzodiazepine; ↑ ArTH	No consistent AHI reduction alone	Adjunct only (selected)	[61]

Effects are pooled or representative values from the cited RCTs and meta-analyses. *AHI, apnea–hypopnea index; CA, carbonic anhydrase; ArTH, respiratory arousal threshold; SARI, serotonin antagonist and reuptake inhibitor*. ↑, increase; ↓, decrease.

**Table 5:** Wake-promoting agents for residual excessive daytime sleepiness in OSA.

Agent	Mechanism	Evidence/Effect	Status	Reference
Solriamfetol	DNRI (no monoamine release)	Dose-dependent ↑ MWT and ↓ ESS (TONES 3)	FDA/EMA approved for EDS	[66]
Pitolisant	H_3_-receptor inverse agonist	↓ ESS in CPAP-adherent and CPAP-refusing OSA	Approved (EU)	[67,68]
Modafinil/armodafinil	Wake-promoting (dopaminergic/noradrenergic)	↑ wakefulness; no AHI effect	EU authorization for OSA withdrawn	[9,65]

*EDS, excessive daytime sleepiness; DNRI, dopamine–norepinephrine reuptake inhibitor; ESS, Epworth Sleepiness Scale; MWT, Maintenance of Wakefulness Test; CPAP, continuous positive airway pressure.* ↑, increase; ↓, decrease.

**Table 6:** Principal adverse effects, cautions and contraindications, and suggested monitoring for the drug classes discussed.

Drug Class (Examples)	Principal Adverse Effects	Cautions and Contraindications	Suggested Monitoring
Incretin-based therapy (tirzepatide, semaglutide, liraglutide)	Nausea, vomiting, diarrhea, constipation; cholelithiasis; loss of lean as well as fat mass (possible sarcopenia in older patients); weight regain after discontinuation	Personal or family history of medullary thyroid carcinoma or MEN2; prior pancreatitis; severe gastroparesis; pregnancy	Body weight and, where possible, body composition; gastrointestinal tolerance during dose escalation
Noradrenergic–antimuscarinic (AD109, atomoxetine–oxybutynin)	Dry mouth, insomnia, nausea, urinary hesitation, constipation; resting heart rate increased by ~7 bpm; adverse-event-related discontinuation 21.2% in SynAIRgy	Narrow-angle glaucoma; bladder outlet obstruction; uncontrolled hypertension or tachyarrhythmia; monoamine oxidase inhibitor use	Heart rate and blood pressure; urinary symptoms; sleep quality in the first weeks
Carbonic anhydrase inhibitors (acetazolamide, sultiame)	Paresthesia (dose-dependent; up to 57% at sultiame 300 mg), dysgeusia, metabolic acidosis, hypokalemia, polyuria, nephrolithiasis, fatigue	Sulfonamide hypersensitivity; renal or hepatic impairment; pre-existing hyperchloremic acidosis; concurrent high-dose salicylate	Serum electrolytes and bicarbonate; renal function; symptoms of paresthesia
Sedative–hypnotics (trazodone, eszopiclone, sodium oxybate)	Next-day sedation; falls and fractures in older adults; cognitive impairment; dependence (z-drugs); orthostatic hypotension and priapism (trazodone); sodium load and respiratory depression (oxybate)	Age > 65 with fall risk; concomitant opioids or alcohol; hypercapnic respiratory failure or obesity hypoventilation; heart failure or uncontrolled hypertension (oxybate sodium load)	Daytime alertness and driving safety; fall risk; blood pressure; nocturnal oxygenation and CO_2_ where indicated
Wake-promoting agents (solriamfetol, pitolisant)	Headache, nausea, insomnia, reduced appetite; increased blood pressure and heart rate (solriamfetol); QT prolongation (pitolisant)	Uncontrolled hypertension or recent myocardial infarction/arrhythmia (solriamfetol); severe hepatic impairment or concomitant QT-prolonging drugs (pitolisant)	Blood pressure and heart rate; ECG where clinically indicated
Supplemental oxygen (not a pharmacological agent)	Prolongation of apneas; CO_2_ retention in susceptible patients	Hypercapnic respiratory failure; obesity hypoventilation syndrome	Daytime and nocturnal CO_2_; event duration on follow-up study

*MEN2, multiple endocrine neoplasia type 2; bpm, beats per minute; ECG, electrocardiogram.*

## Data Availability

No new data were created or analyzed in this study. Data sharing is not applicable.
